# Nanoantioxidants: Pioneer Types, Advantages, Limitations, and Future Insights

**DOI:** 10.3390/molecules26227031

**Published:** 2021-11-21

**Authors:** Basma Omran, Kwang-Hyun Baek

**Affiliations:** 1 Department of Biotechnology, Yeungnam University, Gyeongsan 38541, Gyeongbuk, Korea; obasma@ynu.ac.kr; 2 Department of Processes Design & Development, Egyptian Petroleum Research Institute (EPRI), Cairo 11727, Egypt

**Keywords:** oxidative stress, nanoantioxidants, nanotoxicity, nanoencapsulation

## Abstract

Free radicals are generated as byproducts of normal metabolic processes as well as due to exposure to several environmental pollutants. They are highly reactive species, causing cellular damage and are associated with a plethora of oxidative stress-related diseases and disorders. Antioxidants can control autoxidation by interfering with free radical propagation or inhibiting free radical formation, reducing oxidative stress, improving immune function, and increasing health longevity. Antioxidant functionalized metal nanoparticles, transition metal oxides, and nanocomposites have been identified as potent nanoantioxidants. They can be formulated in monometallic, bimetallic, and multi-metallic combinations via chemical and green synthesis techniques. The intrinsic antioxidant properties of nanomaterials are dependent on their tunable configuration, physico-chemical properties, crystallinity, surface charge, particle size, surface-to-volume ratio, and surface coating. Nanoantioxidants have several advantages over conventional antioxidants, involving increased bioavailability, controlled release, and targeted delivery to the site of action. This review emphasizes the most pioneering types of nanoantioxidants such as nanoceria, silica nanoparticles, polydopamine nanoparticles, and nanocomposite-, polysaccharide-, and protein-based nanoantioxidants. This review overviews the antioxidant potential of biologically synthesized nanomaterials, which have emerged as significant alternatives due to their biocompatibility and high stability. The promising nanoencapsulation nanosystems such as solid lipid nanoparticles, nanostructured lipid carriers, and liposome nanoparticles are highlighted. The advantages, limitations, and future insights of nanoantioxidant applications are discussed.

## 1. Introduction

**Figure 1:**
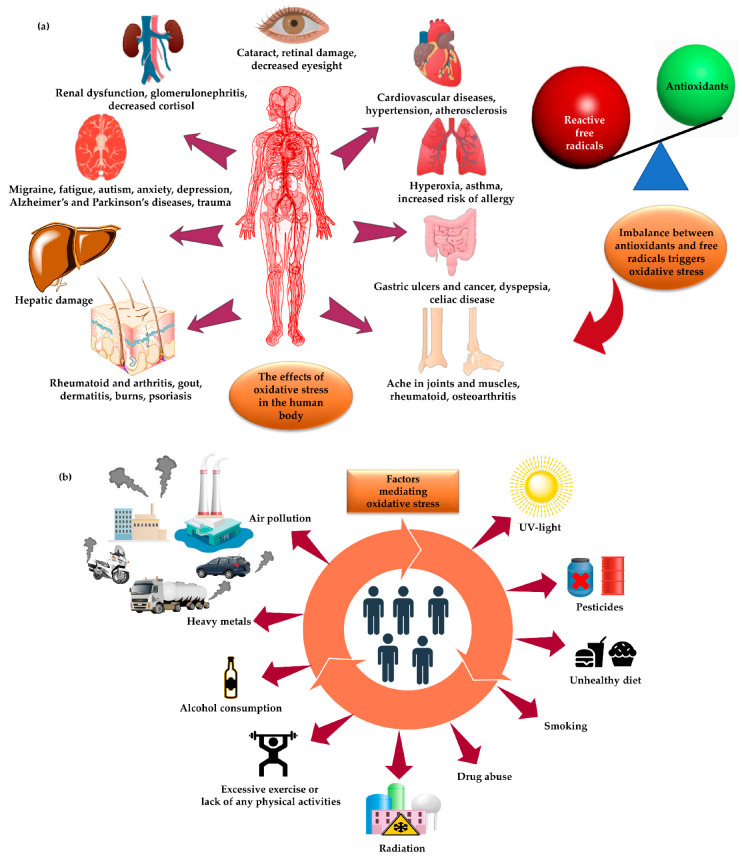
Exposure to different toxic chemicals triggers oxidative stress progression (**a**) and the incidence of oxidative stress in the human body is accompanied by a plethora of pathological diseases and disorders that cause damage to the heart, lungs, intestine, joints, muscles, skin, liver, brain, kidneys, eyes, and immune system (**b**).

Environmental pollution, climatic changes, and unhealthy lifestyles have increased the prevalence of serious and chronic diseases and age-related disorders [1]. Evidence from toxicological investigations on cultured cells and animal models has shown a strong and direct relationship between exposure to hazardous pollutants and generation of oxidative stress, leading to the development of a plethora of pathological diseases such as renal failure [2], atherosclerosis [3], diabetes [4], systemic inflammation [5], acute respiratory syndrome [6], asthma [7], Alzheimer’s and Parkinson’s disorders [8], certain types of cancer [9], and arthritis [10], etc. (Figure 1). In 1985, the concept of oxidative stress was formulated by Helmut Sies in the book entitled “Oxidative Stress” to describe the disruption of the balance between antioxidative defenses and oxidant molecules [11]. The imbalance between oxidants and the cellular defensive antioxidants triggers oxidative stress. In 1956, the term ‘reactive species” was coined to refer to oxidizing radicals [12].

The reaction of antioxidants with free radicals is governed by various mechanisms, involving hydrogen atom transfer (HAT), single electron transfer (SET), and proton-coupled electron transfer mechanisms [13,14]. The HAT mechanism is based on the ability of antioxidants to quench free radicals by detaching a phenolic hydrogen atom from the antioxidant via homolytic cleavage of the O–H bond to the free radical, producing a stable product [15]. In this mechanism, the antioxidant is converted into a radical, which is significantly more stable than the original free radical, and as a result, inhibiting the overall oxidation processes [16]. The bond dissociation enthalpy (BDE), a numerical parameter related to HAT, is an essential parameter to evaluate the antioxidant activity in this mechanism [17]. Generally, the lower the BDE of the H-donating group of the antioxidant, the easier it is to inactivate free radicals, and thus the high is the antioxidant activity of a compound [18]. Examples of HAT-based assays are the oxygen radical absorption capacity (ORAC), total peroxyl radical trapping antioxidant parameter (TRAP), and total oxyradical scavenging capacity (TOSC) assays [19].

The SET mechanism is based on the transfer of a single electron from the antioxidant to the free radical. The antioxidant molecule is converted to an intermediate radical cation that is less reactive than the original free radical [20]. In the SET mechanism, the ionization potential (IP) of the reactive functional group of the antioxidant is the most significant parameter for determining its antioxidant activity. Examples of SET-based assays involve cupric reducing antioxidant capacity (CUPRAC), the Folin–Ciocalteu (FC), and the ferric reduction of antioxidant power (FRAP) [19]. SET can be subdivided into single-electron transfer followed by a proton transfer (SET-PT) and sequential proton loss electron transfer (SPLET) [21,22]. In the SET-PT mechanism, an electron is first transferred from the phenolic OH to the radical and the phenolic OH becomes a phenoxy radical cation (PhOH^•+^), which deprotonates during the second step, resulting in the formation of a phenoxy radical (PhO^•^) [23]. In the SET-PT mechanism, the radical transfer (i.e., first stage) is measured as the ionization potential (IP) and the deprotonation (i.e., second stage) corresponds to the proton dissociation enthalpy (PDE). The SPLET mechanism was first proposed by Litwinienko and Ingold [24,25,26]. This mechanism proceeds via two stages, involving (i) deprotonation of PhOH and formation of a phenoxide ion (PhO^−^) and (ii) transfer of an electron from PhO^−^ ion to the radical, leading to the formation of PhO^•^ [27,28]. The enthalpies of the first and second stages in the SPLET mechanism are denoted as proton affinity and electron transfer enthalpy, respectively. The aforementioned mechanisms are dependent on radical and solvent properties [29].

Proton-coupled electron transfer (PCET) is a more complex mechanism by which an electron and a proton are transferred and their coupling has significant thermodynamic and kinetic impacts on the process [30]. In the PCET mechanism, the transfer of an electron and a proton can be sequential or concerted. In the PCET sequential transfer mechanism, the electron can be transferred first and this process is designated as electron transfer-proton transfer (ETPT). On the other hand, when the proton is transferred first, the process is referred to as proton transfer-electron transfer (PT-ET). In the concerted PCET mechanism, the electron and the proton are transferred simultaneously [31].

Reactive oxygen species (ROS) include radical species such as hydroxyl radical (^•^OH) and superoxide anion radical (O_2_^•−^), while singlet oxygen (^1^O_2_), hydrogen peroxide (H_2_O_2_), ozone (O_3_), lipid peroxide (LOOH), and hypochlorous acid (HOCl) represent the non-radical forms of ROS [32]. Reactive nitrogen species (RNS) include nitric oxide (^•^NO), peroxynitrite (ONO_2_^−^), nitrous oxide (N_2_O), nitrogen dioxide (NO^•^_2_), peroxynitrous acid (HNO_3_), and nitroxyl anion (NO^−^) radicals [33]. Reactive oxygen and nitrogen species (RONS) are spontaneously produced by endogenous metabolic pathways, for instance the electron transport chain (ETC) or by enzymes like xanthine oxidase and nitric oxide synthase. However, excessive exposure to some exogenous factors such as ionizing radiation, heavy metals, and UV radiation also trigger the generation of excessive levels of RONS [34]. Under homeostatic conditions, the thresholds of RONS are tightly controlled at a systemic level to fine-tune multiple physiological processes, including autophagy, immune and mitogen induction, muscle contraction, enzymatic activities, and cell signal transduction pathways [35]. Nevertheless, the sustained increase of free radical levels (e.g., ROS and/or RNS) suppresses the natural body defense mechanisms, causing significant damage to biological macromolecules, leading to aberrant protein expression, lipid peroxidation, oxidative DNA disruption, red blood cell degradation, enzyme inactivation, and vascular and nerve impairment, hence affecting normal cellular functions [36].

Human cells possess cellular mechanisms devoted to the establishment of redox homeostasis. The disruption of this homeostasis has substantial pathophysiological implications [37]. In human cells, endogenous enzymatic and non-enzymatic defense mechanisms counteract the disruption triggered by oxidative stress and help maintain appropriate levels of RONS [38]. The enzymatic defenses involve catalases (CAT), superoxide dismutases (SOD), and glutathione peroxidases (GSH-Px), whereas the non-enzymatic defenses involve glutathione, uric and lipoic acids, thioredoxin, bilirubin, and ubiquinone [39]. These enzymatic and non-enzymatic molecules have a crucial role in maintaining adequate levels of RONS. Generally, the activity of antioxidants takes place via two basic pathways, either by scavenging RONS or by inhibiting the synthesis of RONS. In certain cases, antioxidants particularly, enzymatic compounds, exhibit antioxidant capacity via the simple degradation of RONS to less damaging intermediates [40]. Exogenous natural antioxidants such as vitamins, carotenoids, polyphenolic compounds, curcumin, and metabolic sensitizers, which are derived from fruits, vegetables, and their byproducts have displayed a high potential to scavenge and combat the exacerbated levels of free radicals [41]. However, natural antioxidants can be degraded prior to delivery to target sites and their biodistribution is restricted by low absorption [42]. Some synthetic antioxidants such as butylated hydroxyanisole, hydroxytoluene, and gallic acid esters have exhibited antioxidant potency; however, adverse health effects limit their use [41].

Nanotechnology has opened up new horizons to explore creative solutions for the treatment of oxidative stress-related disorders. Progress in nanotechnology has revolutionized the potential use of a number of nanomaterials as effective nanoantioxidants [43,44,45,46]. Nevertheless, the antioxidant capacity of these nanostructures varies according to their chemical configuration, nature, surface charge, crystallinity, particle size, and surface coating [47]. Recently, engineered nanoparticles (NPs) and nanocomposites have been considered as nouveau tools for the development of innovative nanoantioxidants with enhanced properties for health longevity. The integration of nanoscience with biomedicine for the development of nanoantioxidant therapy has witnessed a significant breakthrough, leading to major progress in the pharmaceutical and biotechnological industries [48]. Indeed, nanoscience has provided promising solutions to address the challenges associated with conventional antioxidant compounds and to enable the development of pioneering classes of nanoantioxidants [49]. More interestingly, many categories of nanoantioxidants have potent radical scavenging and quenching capacities that have shown greater antioxidative sturdiness and more resistance to severe microenvironments than natural antioxidants. Nanoantioxidants improve the pharmacokinetics of natural antioxidant molecules by preventing their rapid degradation under stress conditions either through nanoencapsulation or nanodelivery [50]. Although antioxidant-functionalized nanostructures have displayed tremendous potential in animal experiments, clinical studies in humans remain elusive.

**Figure 2:**
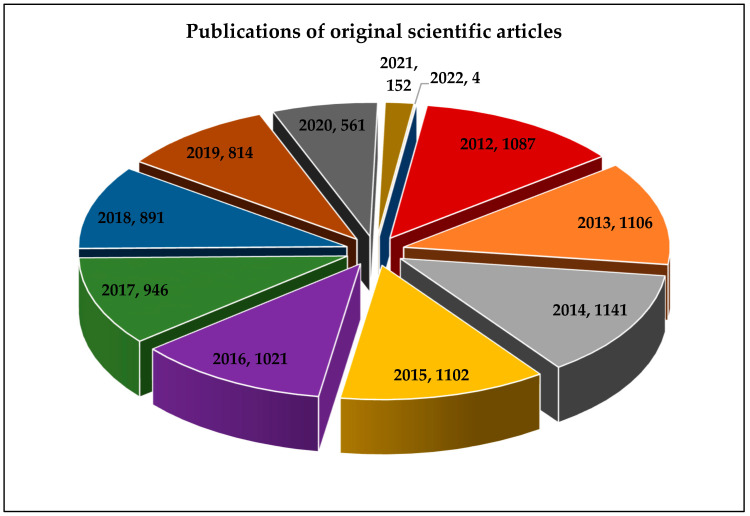
Published original scientific articles under the keywords “nanoantioxidants”, “antioxidant nanomaterials”, and “nanomaterials and antioxidant activity” (based on a SciFinder search; duplicates removed, November 2021).

Reviews describing nanoantioxidant types and diverse applications can be found in the recent scientific literature [51,52,53,54,55]. The presented 3D pie chart illustrates the number of published original scientific literature under the keywords “nanoantioxidants”, “antioxidant nanomaterials”, and “nanomaterials and antioxidant activity” in the last ten years (Figure 2). This review covers the literature published over the last ten years by providing a discussion of the most pioneering nanoantioxidant types, such as ceria NPs, silica NPs, polydopamine NPs, carbohydrate-and protein-based NPs, and nanocomposite-based nanoantioxidants. The promising antioxidant role of biologically synthesized nanomaterials as alternative candidates is also overviewed. Solid lipid NPs, nanostructured lipid carriers, and liposome NPs are overviewed as efficient nanoencapsulation nanosystems. The toxicity concerns of nanoantioxidant applications are emphasized. The advantages, limitations, and future perspectives of nanoantioxidant applications are proposed.

## 2. Pioneer Types of Nanoantioxidants

Antioxidants are molecules that can counteract the action of reactive species and inhibit the subsequent chain reactions before essential biological macromolecules are damaged and cellular processes are disrupted. For a compound to be an antioxidant, it should be capable of forming new intermediates that are non-reactive and quite stable when exposed to further oxidation [56]. Antioxidants are divided into two main classes, including (i) radical-trapping antioxidants, also referred to as chain-breaking antioxidants, which capture chain-carrying radicals and hence cause the breaking of the oxidation chain and (ii) preventive antioxidants, which reduce the rate of radical chains [57]. The antioxidant mode of action involves (i) combating reactive radicals that induce peroxidation, (ii) preventing the generation of reactive radicals and decomposition of peroxides by metal ion chelation, (iii) scavenging O_2_^•−^ to inhibit peroxide production, (iv) interrupting auto-oxidative reactions, and/or (v) minimizing localized O_2_ concentrations [58].

**Figure 3:**
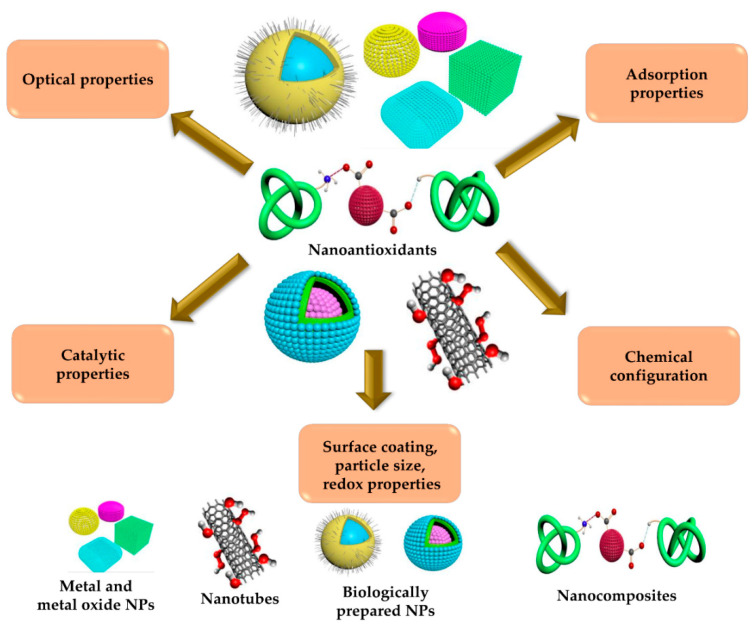
Antioxidant activity of nanomaterials is dependent on their unique properties.

Currently, nanotechnology is growing rapidly in all scientific disciplines. The convergence of nanotechnology with other scientific fields such as biomaterial science, molecular biology, and medicine has contributed to the expansion and progress of nanomedicine. Nanotechnology enables the synthesis and manipulation of nanometer-scale materials, resulting in the development of novel nanomaterials for therapeutic and diagnostic applications in biological systems. NPs are the building blocks of nanoscience and have outstanding properties such as a high surface-area-to-volume ratio [59]. The high crystalline surface area provides NPs with superb mechanical, optical, magnetic, catalytic, and optical properties, thus increasing their potential pharmacological and biomedical applications [60]. Nanoantioxidants are nanomaterials that can slow the overall rate of autoxidation by trapping chain-carrying radicals or reducing initiation processes [61]. The term nanoantioxidant (artificial antioxidants) was proposed by Sharpe et al. [40]. These artificial antioxidants can be engineered at the nanoscale level, depending on the chemical composition, surface charge, surface-to-volume ratio, and surface coating of the NPs [40]. Antioxidants were defined by Halliwell as “any substance that can significantly delay or prevent oxidation of an oxidizable substrate, when present at low concentrations compared to those of that substrate” [56]. Numerous approaches have been established for the synthesis of nanoantioxidants. The most common techniques applied for the preparation of nanoantioxidants are summarized in Table 1. The antioxidant potential of nanoantioxidants can be ascribed to their tunable catalytic and redox properties and the capacity to oscillate between different oxidation states [51] (Figure 3).

**Figure 4:**
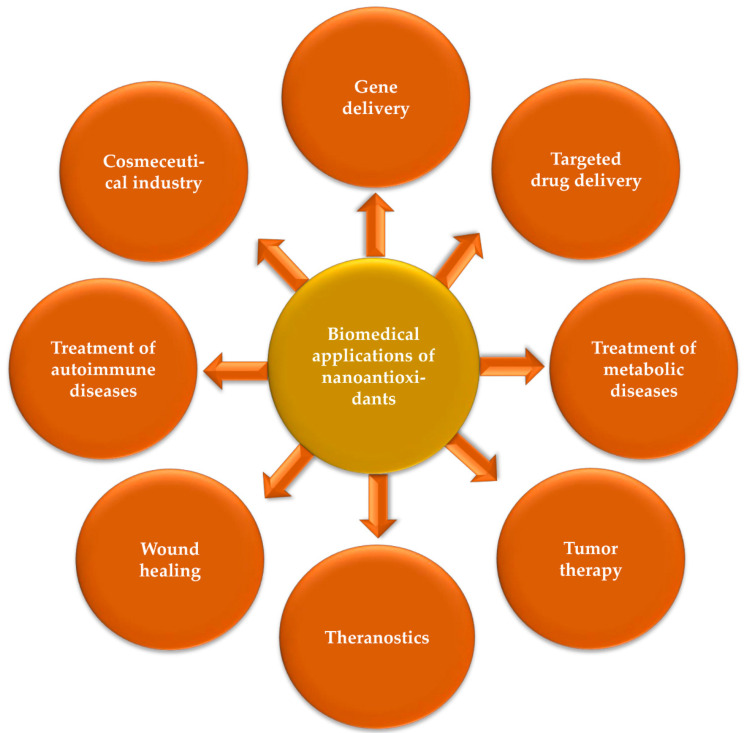
A schematic representation of some of the biomedical applications of nanoantioxidants.

Nanoantioxidant biomedical applications have significantly advanced the biotechnological and pharmaceutical industries, with remarkable progress in the field of antioxidant therapy [64]. Nanoantioxidants have shown promising results to address the challenges associated with conventional antioxidants and have enabled the development of an innovative class of antioxidants [49]. They exhibit high antioxidative potential and high tolerance to harsh microenvironments. The potential use of nanoantioxidants has been reported for several biomedical applications, such as treatment of autoimmune and metabolic diseases [65,66], wound healing [67], tumor therapy [48], drug delivery [68], gene delivery [69], theranostics [70] (Figure 4). The subsections that follow describe the most common and pioneer types of nanoantioxidants based on the previously published literature.

### 2.1. Metal Oxide-Based Nanoantioxidants

Metal oxide NPs are highly reactive particles due to the presence of atoms with unpaired valence electrons located on the NP surface [71]. As a result of the unique characteristics of NPs, their clinical application has significant advantages as compared to conventional treatment therapies, which have several side effects and reduced efficiency at target sites. Metal oxide NPs have been studied for a variety of biomedical applications, involving antioxidant and antimicrobial applications, bioimaging, drug delivery, and biosensing [72].

#### 2.1.1. Nanoceria or Cerium Oxide-Based Nanoantioxidants

**Figure 5:**
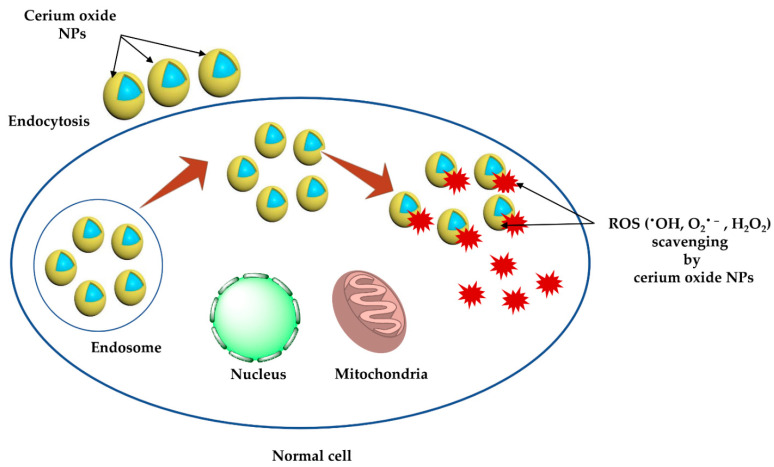
Antioxidant effect of cerium oxide NPs in normal cell under physiological pH via endocytosis and then scavenging ROS, such as ^•^OH, O_2_^•−^, and H_2_O_2_ as a result of SOD mimetic activity, by which O_2_^•−^ is reduced to H_2_O_2_ and CAT activity by which H_2_O_2_ is further degraded into H_2_O, and hence providing protection to normal cells.

Several synthesis techniques have been reported for the chemical fabrication of cerium oxide NPs (CeO_2_ NPs) or nanoceria including chemical precipitation [71], co-precipitation [73], sonochemical [74], sol-gel [75], microwave [76], hydrothermal [77], and microwave-assisted hydrothermal [78] techniques. CeO_2_ NPs are one of the most intriguing metal oxide NPs, which have been widely employed for nanomedical and nano pharmaceutical research fields. Numerous studies have shown that CeO_2_ NPs have the potential to suppress oxidative stress-related diseases such as Alzheimer’s disease [79,80], cardiomyopathy [81], and cancer [82]. Free radicals play a dual role in living cells, having either beneficial or toxic effects. At low/moderate levels, free radicals have several physiological activities, involving immune function, cellular signaling pathways, mitogenic responses, redox signaling, and growth regulation [83]. However, high levels of ROS/RNS can cause oxidative stress and nitrosative stress, respectively, causing significant damage to cellular biomolecules [84]. The ability of CeO_2_ NPs to scavenge free radicals is attributed to their superior optical and catalytic properties and their potential to switch the oxidation states (e.g., Ce^3+^ and Ce^4+^) [85] (Equations (1)–(3)) (Figure 5).

(1) $${\mathrm{SOD}\;\mathrm{mimic}\;\mathrm{activity}\;\mathrm{Ce}}^{4+}+{\;O}_{2}^{•-}\to {\;\mathrm{Ce}}^{3+}+{\;O}_{2}$$

(2) $${\mathrm{Ce}}^{3+}+O_{2}^{•-}+2H^{+}\to {\;\mathrm{Ce}}^{4+}+H_{2}O_{2}$$

(3) $${\mathrm{CAT}\;\mathrm{mimic}\;\mathrm{activity}\;H}_{2}O_{2\;}\to {\;\mathrm{Ce}}^{3+}+2H^{+}\;\to {\mathrm{Ce}}^{4+}+2H_{2}O$$

However, CeO_2_ NPs are poorly soluble in water, posing several challenges when it comes to biological applications. As a result, several studies reported that CeO_2_ NPs could be well-dispersed in aqueous solutions when coated with dextran [86], polyethylene glycol (PEG) [87], and polyacrylic acid [88] to improve their stability, biocompatibility, and water solubility. Impregnation of nanoceria in polymeric matrices such as three dimensional (3D) scaffolds or polymer coatings is a promising option for increasing biocompatibility, but it may result in a reduction in therapeutic effects [89]. For instance, fructans are poly-fructose molecules that are made up of fructose-linked polymers, involving levan and inulin. Levan is a β-2, 6-conjugated fructose polymer. By applying a one-pot co-precipitation technique, CeO_2_ NPs were conjugated with levan that acted as a reductant and a stabilizing agent [90]. Coating CeO_2_ NPs with levan boosted its antioxidant potential. Additionally, ROS levels were reduced in H_2_O_2_-induced NIH3T3 cells after treatment with levan-coated CeO_2_ NPs, which could be useful in the treatment of ROS-mediated diseases. Using 3D printing strategies may also be advantageous for the proper design of structures containing nanoceria to fine-tune their release for better therapeutic effects [89]. Entrapment within an encapsulation hydrogel was reported as a strategy to reduce the cytotoxicity of CeO_2_ NPs. This delivery strategy allows particles to be targeted at the site of interest, reducing phagocytosis while retaining their catalytic potential. A nanocomposite was engineered by the incorporation of CeO_2_ NPs within an encapsulating alginate microbead hydrogel [91]. The as-prepared nanocomposite provided high antioxidant potential and cryoprotection to beta cells against O_2_^•−^ attacks without cytotoxic effects up to 10 mM of encapsulated CeO_2_ NPs. In contrast, phagocytosis of beta cells occurred even at concentrations as low as 1 mM of non-encapsulated CeO_2_ NPs.

Several factors affect the antioxidant potential of nanoantioxidants, such as size, shape, surface charge, agglomeration, coating, and dissolution [92]. The effect of shape and agglomeration on the antioxidant activity of CeO_2_ NPs-modified with AuNPs was studied [93]. Au/CeO_2_ NPs were prepared by hydrothermal technique and three morphological structures of Au/CeO_2_ NPs were obtained (i.e., NPs, nanocubes, and nanorods). At low concentrations, the antioxidant capacity of Au/CeO_2_ nanorods and NPs was enhanced, while that of Au/CeO_2_ nanocubes was reduced. On the other hand, at high concentrations, a reduction in the antioxidant activity of Au/CeO_2_ nanorods and NPs was observed, while that of Au/CeO_2_ nanocubes was improved. This could be attributed to the severe agglomeration of Au/CeO_2_ nanorods and NPs at high concentrations, which covered the active sites and reduced the antioxidant activity when compared to Au/CeO_2_ nanocubes, which exhibited less aggregation. The behavior of nanocubes was contradictory to that of the NPs and nanorods, which could be attributed to the (1 0 0) crystal planes, which might have had an impact on the transfer of electrons at the interface.

#### 2.1.2. Other Metal Oxide-Based Nanoantioxidants

Coating or surface functionalization of iron oxide (IO) NPs boosts their antioxidant potential [54]. Gallic acid-functionalized Fe_3_O_4_ NPs with average sizes of 5 and 8 nm had 2- to 4-folds lower half maximal inhibitory concentration (IC_50_) values than the non-functionalized Fe_3_O_4_ NPs using the 1, 1-diphenyl-1-picrylhydrazyl (DPPH) scavenging assay [47]. The free radical scavenging activity was due to the reaction of gallic acid with DPPH-radicals. FTIR clearly demonstrated the disappearance of the hydroxyl groups of gallic acid. In another study, under physiological conditions, monocrystalline and orthorhombic forms of vanadia (V_2_O_5_) nanowires with a size of ~100 nm exhibited significant antioxidant potential by mimicking the antioxidant behavior of the antioxidant enzyme GPx [94]. Although the bulk form of V_2_O_5_ is known for its cellular toxicity, this can be dramatically altered when it is reduced to the nanoscale dimensions. Furthermore, the biocompatibility of V_2_O_5_ nanowires may inspire its therapeutic potential for the prevention of oxidative stress-related diseases. Another study showed that 20 nm-sized molybdenum trioxide (MoO_3_) NPs had an intrinsic biomimetic sulfite oxidase potential, allowing for the conversion of sulfite to sulfate and thus had a significant role in the detoxification processes [95]. MoO_3_ NPs were surface-functionalized with dopamine as an anchor group and triphenylphosphonium ion as a targeting agent. Lutetium oxide NPs (Lu_2_O_3_ NPs) were synthesized via the sol-gel technique and doped with europium (Eu^3+^) [96]. The antiradical activity of Lu_2_O_3_ NPs-doped with Eu^3+^ ions remarkably reached 86% as determined by the 2, 2′-azino-bis-3-ethylbenzothiazoline-6-sulfonic acid (ABTS) assay. In another report, copper oxide NPs (CuO NPs) were synthesized using the co-precipitation technique and were coated with PEG and polyvinyl-pyrrolidone (PVP) by simple adsorption [97]. Interestingly, the antioxidant potential of the coated CuO NPs (i.e., CuO-PEG and CuO-PVP NPs) was higher than that of the uncoated particles. Similar behavior was noticed with that of ZnO NPs that were capped with PEG and PVP [98]. The high antioxidant potency could be attributed to the presence of ester linkages and carbonyl groups in both PEG and PVP [99].

### 2.2. Mesoporous Silica or Silicon Dioxide-Based Nanoantioxidants

According to the Woodrow Wilson International Center for Scholars, silica NPs (SNPs) are one of the five most commonly used nanomaterials in consumer products [100]. The US Food and Drug Administration (FDA) approved the use of Cornell Dots (C-Dots) and silica-integrated NPs for clinical trials on humans [101]. SNPs are perfect candidates to overcome a variety of medical problems, including rapid hydrolysis, enzymatic degradation, mass transport complications, difficulties of suspension formulation, and toxicity [102]. SNPs have distinct properties, involving tunable mesoporous structures with versatile morphological structures depending on the synthesis techniques. SNPs are distinguished by their biocompatibility and ease of fabrication, which allow for tunable surface modification [103]. The amorphous structure, size, stability, porosity, and surface modification are dependent on the reaction parameters [104]. For the preparation of gel-type SNPs, the sol-gel technique is usually applied, in which temperature, catalyst concentration, reagent ratio, and pressure are important criteria that can alter the properties of SNPs. Mesoporous SNPs are excellent nano vehicle candidates for smart drug delivery because of their porous structure, which can encapsulate therapeutic agents [105]. Furthermore, altering the surface of the SNPs or incorporating magnetic NPs to their cores can allow in vivo targeting to specific sites [106].

Novel mesoporous silica organic-based nanocomposites have been extensively investigated because of their large surface area and pore size, excellent thermal stability, size control potential, monodispersity, and tunable functionalities [107]. The antiradical properties of silicon dioxide (SiO_2_) NPs were assessed via the DPPH assay using UV/Vis and electron paramagnetic resonance (EPR) spectrophotometers [108]. The SiO_2_ NPs were covalently grafted and functionalized with gallic acid (SiO_2-_GA NPs). The DPPH solution exhibited a rapid decolorization of the initial characteristic purple color upon reaction with the SiO_2-_GA NPs. The key advantage of this preparation was the possibility of gallic acid regeneration. DLS data indicated that SiO_2-_GA NPs exhibited a dramatic agglomeration after their interaction with DPPH-radicals, resulting in an approximately six-fold increase in the size of the SiO_2-_GA NPs compared to their original size (180 nm) before the interaction with the DPPH-radicals. In another study, a silica-based nanocomposite was fabricated using the modified Stöber technique via the incorporation of mesoporous poly-(tannic acid) with SNPs (p(TA)- SNPs) [109]. SEM images showed the formation of 300 nm-sized spherical-shaped p(TA)- SNPs. The Folin–Ciocalteu and Trolox equivalent antioxidant capacity assays were used to assess the antiradical potential of p(TA)-SNPs against gallic acid and Trolox standards, respectively. The antiradical potency of p(TA)- SNPs was 140.3 g/mL total phenol content in terms of gallic acid equivalency and 686 mM trolox equivalent/g.

Two biopolymer silica-based nanocomposites, chitosan-SiO_2_ and carboxy methyl cellulose-SiO_2_ (Chi-CMC-SiO_2_) were fabricated and loaded with an aqueous extract of *Larrea divaricata* [110]. SEM micrographs demonstrated the uniform and porous structure of the prepared silica-based nanocomposites. The antiradical activity was assessed using the DPPH scavenging assay and by evaluating the SOD activity. Chi-CMC-SiO_2_ nanocomposite exhibited 3.5-folds higher antiradical capacity when compared with Chi-SiO_2_ nanocomposite. In another report, a synthetic antioxidant fluorescent flavone was anchored onto APTES-modified SNPs via sulfonamide linkage (FMES) [111]. The polydispersed index of FMFS NPs was 443.7 nm and the zeta potential value was 10.2 mV. FMFS NPs displayed a ferric reducing antiradical value of 55.6 ± 0.06 mM of gallic acid equivalent/g, which was almost similar to quercetin [112]. Uniform spherical-shaped quercetin-loaded SNPs (70–140 nm) were prepared by an oil-in-water microemulsion method [113]. The variations in the sizes of the prepared particles were attributed to the increased concentrations of quercetin. Nitroblue tetrazolium reduction method was used to quantify the generated O_2_^•−^ by xanthine/xanthine oxidase (XO). Quercetin-loaded SNPs displayed a scavenging activity of 73% towards O_2_^•−^, which could be attributable to the highly porous structure of SNPs that provided a favorable platform for increasing the biological activities of the embedded components.

### 2.3. Nanocomposite-Based Nanoantioxidants

Nanocomposites are innovative nanostructures consisting of organic and inorganic components, which act as nanofillers dispersed in ceramic, metal, or polymeric matrices [114]. Nanocomposite configuration depends on the integration of specific matrices with nanofillers such as NPs, nanofibers, or nanofragments that bind together and surround the matrix. Nanocomposites have several phases and at least one, two, or three phases are within the nano dimensional range [115]. The fabrication of nanocomposites has been extensively investigated owing to their distinctive features, novel design, cost-effectiveness, and ease of preparation. The combination of a nanofiller with specific matrices boosts the unique features of the nanocomposite, such as high mechanical stability (i.e., toughness, strength, flexibility), good optical characteristics, low water and gas permeability, and high electrical and thermal conductivity [116]. The properties of nanocomposites are highly dependent on the strength of the matrix and the reinforcing properties of the fillers. The type, size, concentration, and shape of the embedded fillers within the matrix impart unique properties to each nanocomposite [117]. The radical scavenging potential of nanocomposite-based nanoantioxidants is dependent on several criteria such as methods of preparation, used solvents, and nanocomposite size [118]. The primary goal of hybrid nanocomposite-based nanoantioxidants is to introduce innovative nanoantioxidants with multi-functional and innovative properties that are distinct from their individual components.

A nanocomposite-based nanoantioxidant consisting of V_2_O_5_ nanowires and MnO_2_ NPs acted as a platform to mimic the endogenous antioxidant enzyme-based defense reactions [119]. V_2_O_5_ nanowires mimicked the activity of GPx, whereas MnO_2_ NPs mimicked the activities of SOD and CAT. Dopamine was employed as a crosslinker to combine and assemble the two nanomaterials into a nanocomposite form. The prepared V_2_O_5_@PDA@MnO_2_ nanocomposite functioned as a multi-nanozyme model to mimic the intracellular antioxidant enzymes. Both in vitro and in vivo experiments revealed the biocompatibility of the synthesized nanocomposite with superior intracellular ROS scavenging potency, indicating its potential use in inflammation therapies. In another study, a nanocomposite (MoS_2_@TiO_2_) containing TiO_2_ nano-belts and decorated with MoS_2_ NPs was synthesized [120]. The prepared MoS_2_@TiO_2_ nanocomposite had the highest CAT and SOD activities under physiologically relevant conditions. Notably, the nanocomposite activity surpassed the single components in terms of dispersibility and catalytic behavior. This could be attributed to the high surface area and the exposure of more reactive sites. The MoS_2_@TiO_2_ nanocomposite exhibited good in vitro and in vivo biocompatibility as well as efficient elimination and restoration of endogenous ROS to the normal levels using fibroblast cell (L929) line as a testing model. In another study, the ROS-scavenging potency of the CDs-CeO_2_ nanocomposite was assessed using 2′,7′-dichlorofluorescin diacetate dye-based assay (DCFHDA). CDs-CeO_2_ nanocomposite had the potential to scavenge ROS and prevent H_2_O_2_-mediated oxidative stress in NIH3T3 fibroblast cells [121]. The initiation of radical adducts is largely dependent on the sp^2^ carbon content. However, CDs with very little sp^2^ carbon content can display scavenging potency [122].

The stability and dispersibility of IO NPs in aqueous media represent the two main barriers to their biomedical applications. IO NPs have a high tendency for agglomeration due to interactions with the NPs themselves or with other biological molecules, leading to subsequent precipitation out of the colloidal system [123]. Effective approaches have been investigated to enhance the stability of IO NPs either by grafting with polymers or surfactants or by encapsulation with inorganic coating materials, such as metals, non-metals, metal oxides, and/or metal sulfides [124]. It is worth mentioning that surface coating not only prevents IO NPs from being aggregated but also provides them with more functionality. The antiradical potential of a rod-shaped chitosan-coated iron oxide nanocomposite (CS-FeO) (20–90 nm) was evaluated using the DPPH and H_2_O_2_ assays [125]. The scavenging potency was concentration-dependent as it increased with the increase in the nanocomposite concentration. Interestingly, CS-FeO nanocomposites showed higher scavenging activity compared to the uncoated FeO NPs. The free radical scavenging efficiency of CS-FeO nanocomposites might be due to the presence of phenolic molecules that had the ability to release the hydrogen atoms located in their hydroxyl groups. In another report, reduced graphene oxide/zinc oxide nanocomposite-decorated with PdNPs (Pd-RGO-ZnO) was synthesized via a one-pot hydrothermal method [126]. The size of the Pd-RGO-ZnO nanocomposite ranged from 20 to 50 nm. The scavenging activity was concentration-dependent and the highest radical scavenging potential was 48.6% using 200 μg/mL of Pd-RGO-ZnO.

Interestingly, the progress of advanced technologies such as microfluidics enables the development of large-scale production of nanocomposites with desirable characteristics. Microfluidic reactors provide a facility for the reaction of small reagent volumes, precise monitoring of fluid blending, effective mass transport, better heat transfer, safe and low-cost operations, and ease of automation in a short period [127]. They are extremely useful in a variety of applications, including chemical, biological, material, and pharmaceutical industries [128].

### 2.4. PDA-Based Nanoantioxidants

**Figure 6:**
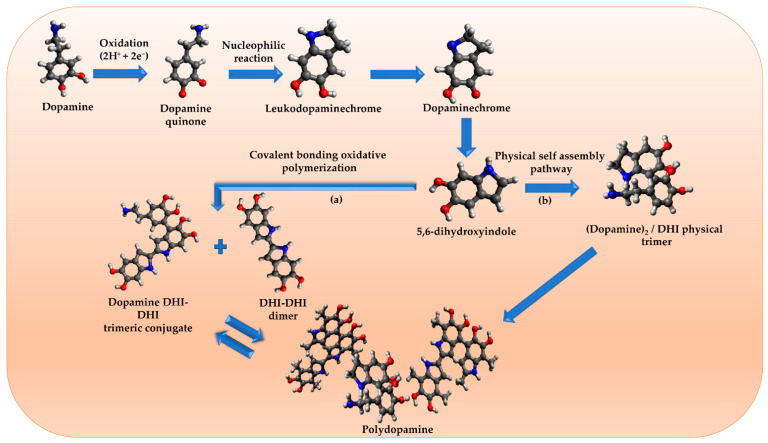
The two pathways applied for polydopamine polymerization, (**a**) covalent bonding oxidative polymerization and (**b**) physical self-assembly.

PDA has recently been studied as a novel biomaterial with numerous biomedical applications such as drug carriers [129]. In 2007, Lee et al. [130] proposed the groundbreaking pathways for PDA polymerization inspired by the exceptional adhesive properties of mussel proteins and their ability to bind to almost any type of surface. The two proposed pathways are the covalent bonding oxidative polymerization as well as physical self-assembly (Figure 6). The adhesive power was attributed to the amino acid, lysine and 3,4-dihydroxy-L-phenylalanine (DOPA) [131]. Dopamine, a DOPA derivative, is a catecholamine that has a significant role in mussel strong adhesion to a variety of substrates via covalent and non-covalent linkages. PDA, a dopamine polymerized product, can adhere to almost any material, such as metal-, ceramic-, and semiconductor-based polymers [132]. PDA is distinguished by its ease of synthesis without the need for organic solvents. Furthermore, adjusting synthesis parameters, such as temperature, pH, reaction time, and oxidant and dopamine concentrations aids in controlling the thickness of PDA films [133]. NPs can be significantly loaded onto PDA via physical or chemical bonds as a result of the abundance of catechol/quinone moieties [134]. The unique properties of PDA can enhance the hydrophilicity of the NPs, provide excellent biocompatibility and ROS scavenging potency, and exhibit appropriate biodegradability. PDA has been extensively used for coating organic and inorganic NPs due to its outstanding adhesive and biochemical properties. PDA-based NPs have sparked the interest of researchers for cancer treatment, tissue repair, antibacterial, antioxidant, and anti-inflammatory applications [135].

PDA core-shell NPs were synthesized by a simple stirring of dopamine hydrochloride in a Tris-HCl buffer saline solution at pH 8.5. Using Schiff base chemical bonding, reduced PDA NPs (rPDA NPs) were integrated into oxidized dextran/chitosan hybrid hydrogel [136]. The rPDA NPs were spherical in shape with a particle size of 240 ± 36 nm. The prepared hydrogel exhibited a wound-healing effect due to its outstanding antioxidant and antibacterial properties. The rPDA NPs were biocompatible and provided protection against oxidative damage. In another study, lipid-coated PDA NPs (L-PDA NPs) were prepared using Stöber method [137]. Transmission electron microscope (TEM) and scanning electron microscope (SEM) revealed the uniform rounded shape of L-PDA NPs with an average size of 170 ± 30 nm. The as-prepared L-PDA NPs had both antioxidant and neuroprotective activities and provided a photothermal conversion platform, hence enhancing the neuronal activity. L-PDA NPs suppressed the accumulation of ROS in different SH-SY5Y, which were prone to pro-oxidative stimulation exerted by tert-butyl hydroperoxide. Moreover, L-PDA NPs suppressed mitochondrial ROS-induced dysfunction and enhanced neurite outgrowth.

**Figure 7:**
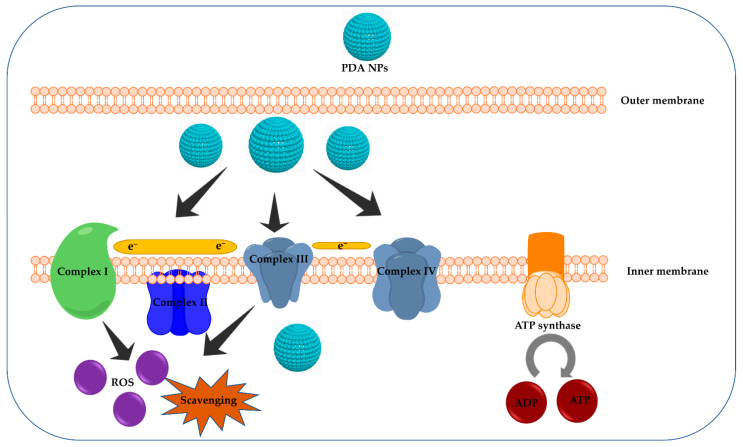
Illustration of PDA NPs as ROS scavengers and as regulators of cellular powerhouses (mitochondria) to reduce osteochondral inflammation.

CuCl_2_ was successfully anchored on PDA-coated magnetic NPs (Fe_3_O_4_@PDA-CuCl_2_), which displayed a uniform and well-dispersed quasi-spherical shape with a mean diameter of 10–20 ± 5 nm [138]. Successful coordination of Cu species on Fe_3_O_4_@PDA surface was confirmed using energy-dispersive X-ray (EDX)-coupled with FESEM, which revealed the presence of elemental peaks of Fe, C, N, O, and Cu. Additionally, TEM images showed the successful coating of dark Fe_3_O_4_ cores with the grey PDA/CuCl_2_ shells. The DPPH radical scavenging activity of the prepared PDA-based nanocomposite was close to that of butylated hydroxytoluene. The development of osteoarthritis has been related to excessive ROS production as a result of mitochondrial dysfunction [139]. In another study, uniform and non-agglomerated PDA nanospheres (117.7 nm) acted as dual-task platforms for the treatment of osteoarthritis by scavenging ROS and regulating cellular powerhouses (Figure 7) [140]. Zeta potential measurements revealed that the NPs were negatively charged (−39.8 mV), which could be ascribed to the abundance of catechol functional groups on the surface of PDA NPs. ROS scavenging activity of PDA NPs was assessed against ^1^O_2_, ^•^OH, H_2_O_2_, and O_2_^•^. A dramatic reduction in ROS levels occurred in presence of different concentrations of PDA NPs. The ^1^O_2_, ^•^OH, H_2_O_2_, and O_2_^•−^ were eliminated using 200 μg/mL of Fe_3_O_4_@PDA-CuCl_2_ with percentages of 66.6%, 71.1%, 76.1%, and 83.9%, respectively, indicating the remarkable scavenging potential and the efficient removal of ROS in aqueous solutions. Furthermore, the intracellular ROS-scavenging potential of PDA NPs was further assessed using DCFHDA and MitoSOX (i.e., mitochondria-specific ROS probe). After incubation with PDA NPs for 24 h, the ROS levels significantly decreased in a PDA concentration-dependent manner. Generally, nutritional molecules are converted to ATP in mitochondria via mitochondrial oxidative phosphorylation (OXPHOS). Mitochondria, cellular powerhouses, are considered significant cellular compartments for ROS production [141]. ROS are generated through the ETC as byproducts of oxidative metabolism. ETC is found in the inner mitochondrial membrane and is comprised of four protein complexes (i.e., Complex I, II, III, and IV) that mediate electron transport and ATP synthesis. To investigate the effect of PDA NPs on mitochondrial functionality, ATP levels were measured in LPS-treated chondrocytes in the presence and absence of PDA NPs. A significant increase in ATP levels was noticed in chondrocytes incubated with LPS-PDA NPs. PDA NPs reversed the decrease in OXPHOS efficiency by boosting the efficiency of Complexes II, III, and IV in the ETC, hence improving mitochondrial function and decreasing mitochondrial ROS production [140].

### 2.5. Polysaccharide-and Protein-Based Nanoantioxidants

Polysaccharide NPs have been extensively studied in a variety of basic and applied science fields, ranging from polymer chemistry to material research to advanced pharmaceutical and biomedical applications [142]. Pharmaceutical applications of polysaccharide NPs involve targeted drug release [143], in vivo drug delivery [144], and stabilizing cosmetic ingredients (e.g., skin and hair care) [145], gene delivery [146], and vaccine delivery [147] as a result of their biodegradability, biocompatibility, muco-adhesive properties, and cellular uptake [148,149]. Cell viability tests have revealed their non-cytotoxicity [150,151], whereas biomedical applications of the polysaccharide NPs involve their use as sensors for the detection of viruses and bacteria [142], tissue engineering [152], wound healing [153], tumor therapy, and delivery of anticancer agents [154].

Chitosan and sodium alginate has been extensively investigated as carrier molecules to encapsulate micro-and nano-particulate drugs under mild conditions [155]. Chitosan (cationic biopolymer) and sodium alginate (anionic biopolymer) have the potential to encapsulate active macromolecular and low molecular weight molecules [156]. Chitosan is a biodegradable and naturally occurring bio-based marine polysaccharide derived from the fractional N-deacetylation of crustacean shell chitin [157]. The poor water solubility of chitosan limits its diverse applications; however, it can be resolved via the preparation of chitosan nanoformulations, which have distinct properties such as antimicrobial and antioxidant activities. In one study, tripolyphosphate acted as a cross-linker for the synthesis of naringenin-encapsulated chitosan NPs (CS-NPs/NAR) via ionic gelation [158]. The free radical scavenging activity of CS-NPs/NAR was evaluated at two concentrations (e.g., 0.3 mg/mL and 0.5 mg/mL). The DPPH and ^•^OH radical scavenging activities were 84% and 75%, respectively using 0.5 mg/mL of CS-NPs/NAR. A significant reduction in nitrate rate (0.16 µM and 0.12 µM) was noticed using 0.3 mg/mL and 0.5 mg/mL of CS-NPs/NAR, respectively. NAR is a potential scavenger of ^•^NO and it reacts with ^•^NO by competing with oxygen, hence aiding in the inhibition of nitrite production. Additionally, NAR released from CS-NPs/NAR had the potential to convert DPPH radicals to non-radical forms, which are inactive. In another report, catechin-loaded NPs were formulated by ionic gelation via the dropwise addition of tripolyphosphate in chitosan cationic solution [159]. Catechin-loaded NPs displayed higher DPPH-radical scavenging activity than pure catechin, exhibiting scavenging activities of 67.01 ± 0.15% and 65.69 ± 0.34%, respectively. Additionally, catechin-loaded NPs exhibited ^•^NO and H_2_O_2_ scavenging activities of 46.12 ± 0.11% and 36.31 ± 0.31%, respectively, which were higher than those of pure catechin, which exhibited scavenging activities of 42.31 ± 0.14% and 38.12 ± 0.11%, respectively. This could be ascribed to the presence of a matrix structure containing ionic polymers. A similar interpretation for the high scavenging activity was proposed in the study of Nallamuthu et al. [160], in which chlorogenic acid, a phenolic compound, was encapsulated into chitosan NPs by ionic gelation method.

Alginate is a naturally occurring biopolymer that is derived from algae, seaweeds, and bacteria [161,162]. It is charcaterized by its high biocompatibility and biodegradability as well as its water-forming gel characteristics. Because of its availability, cost-effectiveness, and ease of formulation, alginate became one of the most commonly used polymers in formulation studies [54]. Alginate nanohydrogels have been used to deliver antioxidants, primarily by combining a multi-functional cross-linker with other polymers to boost the mechanical properties and bioactive release profiles. In one study, AuNPs were synthesized and impregnated with a highly conducting colloidal nanocomposite, polyaniline boronic acid/sodium alginate (PABA-SAL) [163]. The nanocomposite exhibited a moderate antioxidant potential. The highest tested concentration of PABA-SAL@AuNPs nanocomposite (i.e., 6 μg/mL) exhibited a low radical scavenging activity of 15.6%. The results obtained were lower than that of ascorbic acid, a potent antioxidant. Findings obtained from the FRAP assay revealed that both PABA-SAL@AuNPs and ascorbic acid showed the same reduction power.

Proteins are biodegradable, biocompatible, and readily available molecules for NP preparation [164]. Protein-based NPs have been employed for the targeted delivery of bioactive drugs due to their potential to interact with both hydrophilic and hydrophobic moieties [165]. They can act as delivery nano vehicles for DNA, RNA, anticancer drugs, hormones, and growth factors. When compared with other colloidal nanocarriers, protein-based NPs are highly stable and can be easily manufactured using various methods. The most common techniques are desolvation, complex coacervation and nanospray; however, nanospray drying is still in its infancy with some drawbacks, involving decreased flow rates and purity [166]. As a result, more studies are required to resolve these disadvantages. Polycaprolactone-gelatin nanofiber (PGNPNF) mesh-functionalized with CeO_2_ NPs was synthesized by electrospinning and was evaluated for its antioxidative activities in wound healing applications [167]. The synthesized CeO_2_ NPs were ~42 nm in size with a zeta potential value of 30.8 mV. The bioactive PGNPNF nanocomposite maintained the fibrous morphological structure for 14 days. The incorporation of CeO_2_ NPs in the PGNPNF mesh exhibited a SOD mimetic potential. The PGNPNF mesh reduced oxidative stress by ROS scavenging as revealed by relative dichlorofluorescein intensity and fluorescence microscope.

### 2.6. Biologically Fabricated Nanoantioxidants

**Figure 8:**
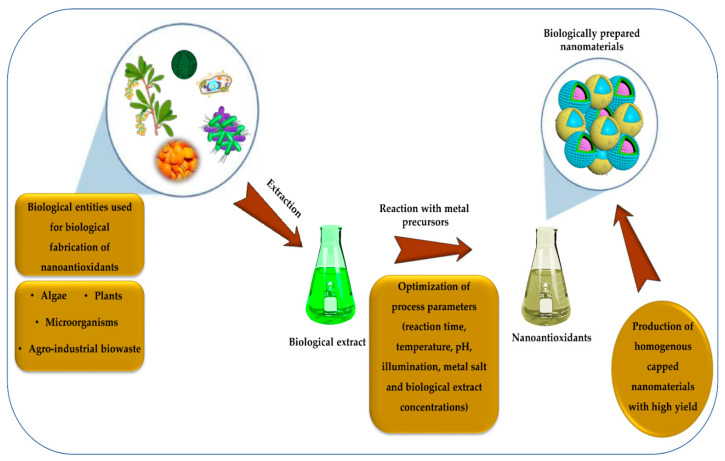
A schematic representation showing the biological fabrication of nanoantioxidants using different biological entities.

The “Green Chemistry” approach first proposed in 1991 was pursued to reduce human and environmental exposure to toxic chemicals as well as decrease or eliminate the use of toxic constituents [168]. In 1998, John C. Warner and Paul Anastas published their pioneer book “Green Chemistry: Theory and Practice” [169]. The twelve key principles of green chemistry included in this book paved the way for inspiring academic scientists and industrial personnel at that time and they continue to influence the advancement of the green chemistry approach to the present day. These fundamental principles affirmed the importance of reducing or eliminating the use of hazardous solvents in chemical reactions. Furthermore, an emphasis was directed towards the urgent need to avoid the release of any toxic waste products or discharges from chemical procedures [170]. The principles shed the light on the importance of using environmentally friendly guidelines during the fabrication, manipulation, and analysis of chemical products. The primary goal of these principles was to decrease any inherent occupational or environmental hazards that may exist while operating industrial activities [171]. Nanobiotechnology has emerged as a significant biological route for NP fabrication that can employ sustainable strategies by following the basic principles of green chemistry [172]. Table 2 shows a list of some of the biologically fabricated monometallic, bimetallic, and metal oxide NPs exhibiting antioxidant/antiradical activity. A schematic representation showing the biological fabrication of nanoantioxidants using different biological entities is illustrated in Figure 8.

Rounded-shaped CeO_2_ NPs with an average diameter of 21 nm were synthesized using the extract of *Stachys japonica* [202]. In vitro studies showed that the *S. japonica*-derived CeO_2_ NPs exhibited an antiradical activity against DPPH-radicals and ABTS^+^ with IC_50_ values of 109.5 ± 0.26 μg/mL and 12.16 ± 0.12 μg/mL, respectively. *S. japonica*-CeO_2_ NPs were biocompatible and efficiently reduced the cellular oxidative stress in mouse fibroblast NIH3T3 cells in terms of cytotoxicity, ROS generation, and prevention of mitochondrial membrane potential (MMP) loss, and nucleus impairment. Neurodegenerative disorders such as Alzheimer’s and Parkinson’s diseases occur as a result of neural cell death and/or weakening of neural cell-to-cell signaling [203]. In a recent study, *Aloe vera* leaf extract acted as a reducing and stabilizing agent for the biological synthesis of 2–3 nm-sized CeO_2_ NPs [204]. Pretreatment of mouse neuroblastoma cells with *A. vera-*derived CeO_2_ NPs resulted in inhibition of oxidative stress in terms of cell viability, generation of intracellular ROS, loss of MMP, neural connectivity, and cell cycle arrest.

Nonetheless, certain drawbacks of green nanoantioxidant fabrication impose further investigations. The most significant disadvantages of the green synthesis of nanoantioxidants are the difficulties in completely separating nanoantioxidants from biomass. On the other hand, the requirement for additional purification stages may have inevitable effects, particularly on potential pilot up-scale production [205]. The antioxidant properties of the biogenic nanomaterials are closely related to the chemical functionalities present on their surface, making standardization difficult. For the efficient synthesis of green nanoantioxidants, it is necessary to optimize the various process parameters that may influence the synthesis of desirable sizes, shapes, and monodispersed particles [206]. To gain a comprehensive understanding of the entire fabrication process, the precise identification of the bioactive molecules participating in the green synthesis of nanoantioxidants is required; however, the presence of numerous phytochemicals and biomolecules makes this a difficult task [205]. The application of synthetic approaches based primarily on the use of sustainable materials is quite interesting and necessitates more in-depth research to achieve commercial and industrial-scale production. Industrial upscaling of green synthesized nanoantioxidants can be accomplished by employing simple, low-cost protocols, and standardizing the synthesis parameters. Biologically fabricated nanoantioxidants have to be precisely fabricated, carefully monitored, and analyzed to overcome challenges related to crystallinity degree, morphological configuration, and conceptual size [207]. More in vivo research on biologically fabricated nanoantioxidants is critical for safe biomedical applications.

## 3. Solid-Lipid-Based NPs, Nanostructured Lipid Carriers, and Liposome NPs as Efficient Nanoencapsulation Systems

### 3.1. Solid-Lipid-Based NPs and Nanostructured Lipid Carriers

**Figure 9:**
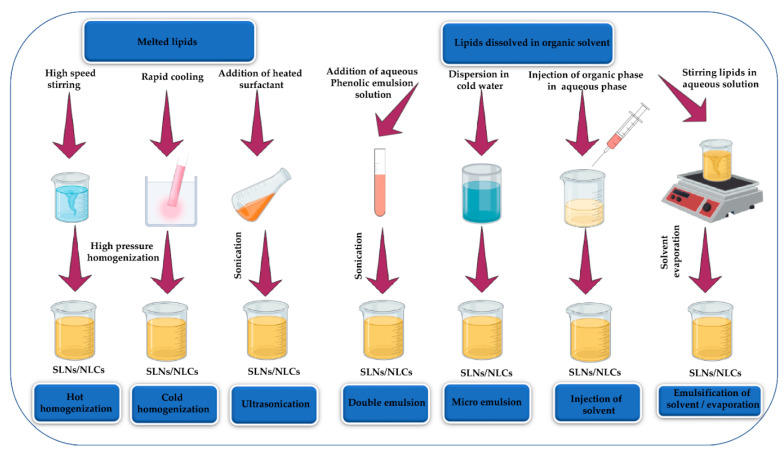
Scheme showing the most common techniques used for the preparation of SLNs and NLCs, involving (i) hot and cold homogenization, (ii) ultrasonication, (iii) double and micro emulsion, (iv) solvent injection, and (v) emulsification/evaporation methods.

The structural configuration of solid lipid nanoparticles (SLNs) and nanostructured lipid carriers (NLCs), as well as their distinguishing features, make them preferable for delivering sensitive bioactive molecules while avoiding chemical degradation. SLNs were first introduced in 1991 as a revolutionary strategy to resolve the various drawbacks associated with other encapsulation methods [208]. SLNs are composed of lipids that remain solid at both the room and human body temperatures, making them extremely useful when it comes to achieving controlled drug release and for the development of new formulations [209]. Moreover, the size of SLNs ranges between 40 and 1000 nm, allowing for a broader range of NPs and bioactive compounds to be encapsulated based on specific requirements [210]. SLNs possess various advantages compared to other colloidal systems, involving high stability, biodegradability, lack of toxicity, the possibility of up-scale production, and the ability of lyophilization, and incorporation of hydrophilic and lipophilic drugs [211]. However, some limitations are associated with SLNs, such as a relatively high water content (i.e., 70%–99.9%), which may represent a major issue during incorporation into a final product [212]. Furthermore, due to the tendency of lipid crystals to recrystallize over time, loading efficiency can decrease. Nanostructured lipid carriers (NLCs) are a new generation of lipid NPs that are made up of both solid and liquid lipids in ratios ranging from 70:30 to 99.9:0.1 [210]. When using liquid lipids, the melting point is reduced, which improves some of the intrinsic properties such as reduced drug release during storage and improved loading potential [213]. However, it is important to note that drug localization in the SLNs/NLCs is also affected by lipophilicity and the structure of the target molecule [214]. For the aforementioned reasons, NLCs are an interesting option for preserving antioxidant molecules while controlling their release rates. Several studies have shown that some antioxidants, such as lycopene [215], quercetin [216], curcumin [217], and astaxanthin [218] can be successfully loaded onto NLCs. Other studies revealed the successful encapsulation of resveratrol [219], idebenone [220], ferulic acid [221], and curcumin [222] on SLNs. Some of the most common techniques applied for the synthesis of SLNs and NLCs are presented in Figure 9 [209].

### 3.2. Liposomes and Their New Generations

Liposomes were first introduced by Alec Bangham in 1961 [223] and since then they have become one of the most common carriers for encapsulation purposes, particularly in cosmetic and pharmaceutical applications. Liposomes are spherical vesicular structures made up of naturally occurring phospholipid bilayers, allowing lipophilic and hydrophilic drugs to be incorporated into the lipid layers and the aqueous core, respectively [224]. Liposomes are characterized by their decreased toxicity, biocompatibility, biodegradability, and non-immunogenicity because of their natural bilayer composition. Liposomes also have the ability to encapsulate hydrophilic, lipophilic, and amphiphilic molecules as well as having a versatile chemical structure [225]. The natural bilayer is composed of phosphatidylcholine, cholesterol, phosphatidylglycerol, dipalmitoylphosphatidylcholine, stearylamine, distearoylphosphatidylcholine, and dicetylphosphat [226]. Properties of liposomes are mainly dependent on their lipid structure, size, surface charge, and method of preparation. The rigidity and the charge of the liposomes are affected by the choice of the bilayer components [227]. Liposome NPs are classified into the following categories based on their size, involving small uni-lamellar vesicle (20–100 nm), large uni-lamellar vesicle (>100 nm), multi-lamellar vesicle (>500 nm), and multi-vesicular vesicle (>1000 nm) [228].

Liposomes as drug delivery systems have become a biomedical research hotspot because they offer specific-targeted delivery at target sites [229]. The innovative use of liposomes has significantly improved therapies by providing stability to therapeutic compounds, tackling the limitations related to cellular and tissue uptake, and enhancing their biodistribution and permeation at target sites in vivo [230]. Drugs loaded into liposomes are further protected from the surrounding physiological conditions, including enzymatic degradation, immunologic inactivation, and rapid plasma clearance, thus improving and extending their action. Liposomes have been reported as efficient nanoencapsulation systems because they can fuse with the cell membrane and deliver the exogenous drug molecules to target cells due to the similarity of the liposome lipid bilayer to the real cellular membrane [231]. However, the use of liposomes can be restricted due to their short life span, sensitivity to oxidation, tendency for aggregation, and poor response to external stimuli [232,233]. Furthermore, one of the major disadvantages of liposome formulation is its rapid clearance from systemic circulation. This rapid clearance is caused by the absorption of the phospholipid membrane that comprises the liposome structure by plasma proteins [234]. To overcome these limitations, liposome–polymer integration is regarded as an innovative strategy for improving in vitro and in vivo performance of liposomes [235]. Liposomes can be modified with polymers either by surface coating or grafting [236]. Liposome surface modification alters their shape, size, surface charge, and lipid chain ordering. Consequently, surface-modified liposomes can exhibit improved biological properties, such as increased stability, consistent targeting, and long-term circulation [237]. Examples of the several biopolymers that can be used for the modification of liposome surface involve chitosan, cellulose, pectin, polysaccharide gums, and proteins [238,239,240,241]. Moreover, coating the liposomal surface with hydrophilic polymers such as polyethylene glycol (PEGs) can slow the clearance of liposomes. PEGs are characterized by low immunogenicity and antigenicity, hence can act as a protective covering over the liposome phospholipid bilayer [234,242]. The improved stability of surface-modified liposomes is due to the electrostatic and/or steric repulsion between liposome vesicles. Various attempts have been carried out to develop new generations of liposome NPs such as transferosomes, ethosomes, niosomes, cubosomes, biosomes, novasomes, and vesosomes. The composition and advantages of each new generation of liposomes are shown in Table 3.

## 4. Toxicity Concerns of Nanoantioxidant Applications

The discipline of nanotoxicology is concerned with determining the toxicity and safety of NMs [251]. The assessment models used for this purpose include 2D cultures at the cellular level, small animal experiments (mice and rats), and traditional model organisms (zebrafish). 2D cultures are basic and beneficial means for toxicity assessment. They have several merits, including low cost, short test period, and the ability to obtain quick results; thus, they can be used to conduct pro-phase experiments under controlled conditions. However, they have drawbacks, such as the inability to simulate the human body’s complex tissue structure and microenvironment. Animal experiments are complicated because of the inevitable species differences as well as ethical concerns that may prevent their use by the scientific community. Recently, some emerging non-animal models have gradually emerged for toxicity assessment, including 3D co-cultures, organoids, as well as organ-on-chip technologies [252,253,254]. These emerging non-animal models can simulate the structure and key functions of human organs. More notably, when compared to 2D cultures, they can reproduce the interactions between cells and secreted substances in the microenvironment. Furthermore, they have other advantages, such as high throughput and less consumption [255].

Although nanoantioxidant-based therapies have shown promising results in laboratories, there are still some hurdles to overcome before their use in clinical applications. In vivo studies are a prerequisite for assessing the biological effects of nanoantioxidants. In vivo systems are distinguished by their complexity, which allows the elucidation of diverse biodistribution and metabolic mechanisms as well as excretion and immune responses [256]. For instance, several in vitro and in vivo animal studies indicated that different shapes and sizes of nanoceria have therapeutic efficacy; however, no clinical trials have certified their use in the treatment of human diseases [89]. This could be attributed to the deficiency of clinical guidelines and care protocols for the use of nanoceria. In this regard, more research is mandatory to thoroughly determine the possible accumulation and clearance of nanoceria in target tissues and vital organs and to define the best administration routes and optimum doses. Attention must be taken into account when using nanoceria because their pharmacokinetics and distribution differ significantly from their bulk counterparts. In male Wistar adult rats, toxicity studies were carried out to evaluate the histopathological changes after the administration of CeO_2_ NPs that were synthesized using pullulan [257]. When compared to negative control groups, in vivo experimental results revealed that CeO_2_ NPs had no significant effects at hematological and biochemical levels. Furthermore, the biosafety of CeO_2_ NPs was evaluated by performing histopathological examinations on kidney and liver tissues using an optical microscope, which confirmed the absence of any significant pathological modifications. Findings were consistent with those reported by Srinivas et al. [258]. There were no negative effects on kidney and liver functions, hematology, or blood biochemistry of the tested rats after the exposure to CeO_2_ NPs in either study. Using the serum of Wistar rats, the pullulan-mediated CeO_2_ NPs displayed a strong anti-radical activity as revealed by ferric reducing/antioxidant power (FRAP) assay. This was due to their ability to bind to oxygen under various oxidizing and reducing conditions. Future research should be directed towards elucidating the long-term fate of nanoceria and quantifying the lowest nanoceria therapeutic doses that can be administered without causing any toxic effects.

One of the major issues causing complications during the interpretation of SNP nanotoxicity is the presence of numerous influential parameters that must be controlled one by one. Nanotoxicity of SNP are influenced by two major factors: (i) physicochemical properties such as size, surface charge, surface area, aggregation severity, porosity, geometry, degradability, density, and dissolution, and (ii) treatment conditions such as dose concentration, tested medium, exposure time, cell type, and animal model [259]. The use of capping molecules to prevent the off-target release of SNPs is an innovative strategy. These capping molecules can be polymeric multi-layered supra-molecules, DNA constructs, or proteins [260]. Fabrication of these complexes facilitates the responses towards the changes of specific stimuli, such as redox chemistry, temperature, pH, and enzymes [102]. The effect of the SNP size administrated intravenously on the biodistribution and pharmacokinetic behavior in mice was investigated [261]. Interestingly, small-sized SNPs (i.e., 50 nm) exhibited no significant toxic effect, whereas large-sized SNPs (i.e., 100 and 200 nm) caused liver inflammation in mice. All SNPs were excreted via urine and bile; however, particles accumulated in the liver and spleen remained for four weeks after the single first dose injection [262]. As a result, biosafety considerations, long-term accumulation in tissues and organs, nanotoxicity, and biodegradability of nano-sized silica particles necessitate further research [263]. Comprehensive research is required to precisely assess the genotoxicity levels and mechanisms, the impact of long-term exposure to SNPs, the effect of their physicochemical characteristics, their routes of exposure, and the role of the immune response [259]. Ecotoxicological studies for assessing the potential risk of SNP are critical for a safer application and handling. To control its potential hazards, safety designs, biological monitoring, appropriate hazard evaluation, and suitable waste discharge should be implemented [264]. As a result of mass production, widespread application, and potential environmental release, occupational exposure to SNPs has become increasingly common and unavoidably resulting in different impacts on human health [265]. Several studies postulated that uncontrolled exposure to SNPs can result in negative impacts on multiple organs and systems, involving respiratory [266], cardiovascular [267], hepatic [268], renal [269], nervous [270], immune [271], and reproductive systems [272].

In one study, in vivo toxicity of CeO_2_ NPs synthesized by a co-precipitation technique via the reaction of cerium nitrate (i.e., precursor) and xanthan gum (i.e., a hydrophilic polysaccharide with cellulose-like biopolymer and a green stabilizer) was investigated [273]. Intraperitoneal injection was applied to assure the uniform distribution of CeO_2_ NPs in the tissues of albino laboratory male rats. The biochemical and histological studies revealed that the green synthesized CeO_2_ NPs exhibited a dose-dependent biological activity. Serum samples were analyzed for some biochemical parameters, including aspartate transaminase, alanine aminotransferase, creatinine, and blood urea nitrogen. The effect of CeO_2_ NPs on the activity of the two antioxidant enzymes (e.g., SOD and CAT), peroxidation of liver lipids, and liver histopathology were also studied. The intraperitoneal administration of CeO_2_ NPs at a dose of 30 mg/kg had no significant changes on the biochemical parameters or antioxidant status, whereas the injection of CeO_2_ NPs at a dose of 60 mg/kg dramatically reduced the activities of serum liver enzyme and enhanced the activities of serum CAT and SOD. However, for long-term biomedical applications, proper dose selection is mandatory. In another study, selenium NPs-enriched polysaccharide (Se-POP-21) synthesized by *Pleurotus ostreatus* were purified, analyzed using column chromatography, and evaluated for their antioxidant potential [274]. Se-POP-21 expressed a strong scavenging effect towards DPPH-radicals and ^•^OH and the scavenging rate was concentration-dependent. Interestingly, Se-POP-21 exhibited no remarkable effects on normal cells.

## 5. Advantages of Nanoantioxidant Applications

Progress in the design of nanomaterials with antioxidant activity over the last few years has given rise to promising therapeutic applications at different target sites [275]. The unique opportunities for clinical use of nanoantioxidants are attributed to the fact that their size is larger than the size of renal filtration (i.e., 10 nm) and can therefore be retained in circulation for longer periods [276]. Nanoantioxidants can also be used at low doses but with high efficacy, minimizing any possible adverse health effects. Nanoantioxidants are characterized by high accessibility, reactivity, and responsiveness to task-specific functions in targeted tissues. Treatment with nanoantioxidants can be integrated with conventional therapy to provide a potential therapeutic option for patients with oxidative stress-mediated pathological disorders. In this context, excellent efficiency has been achieved by covalent linkage and encapsulation of natural antioxidants within nanospheres containing antioxidant-functionalized nanomaterials [277].

## 6. Limitations of Nanoantioxidant Applications

### 6.1. Limitations of Nanoantioxidant Methods of Detection and Measurements

Although nanoantioxidants possess distinct advantages over conventional antioxidants, they do have some limitations [278]. Prior to the clinical use of nanoantioxidants, several critical hurdles and challenges need to be addressed. Spectroscopic or fluorometric detection techniques of nanoantioxidants are influenced by interference, caused by overlaps between the absorption peaks of nanomaterials tested and those of the probes or reaction products. Noble metal-based nanomaterials reveal surface plasmon resonance (SPR) properties at visible wavelengths, which may cause the falsification of absorption peaks. The properties of SPR are usually influenced by the size, shape, and surface configuration of nanoantioxidants [279]. Interference of antioxidant determination assays is significantly related to the types of nanomaterials employed and their catalytic, absorption, fluorescence, and optical properties. Additionally, the suitability of the assay to determine the nanoantioxidant potential should be studied on a case-to-case basis to prevent any incorrect measurements. EPR is also referred to as electron spin resonance (ESR) or electron magnetic resonance (EMR) spectroscopy. The EPR spectrophotometer provides remarkable data for the identification, quantification, dynamics, and analysis of ROS and RNS. EPR spectrophotometer detects free radicals directly and accurately even at extremely low concentrations (i.e., 1 µM) [280]. It is distinguished by the ability to directly measure ROS in vivo. When dealing with short-lived ROS, the spin-trapping technique forms a spin adduct by adding radicals to a nitrone spin, which has a comparatively long half-life and can be detected using an EPR spectrophotometer. EPR combined with appropriate spin traps have emerged as powerful tools for measuring ^•^OH, O_2_^•−^, and ^•^NO in biological systems [280]. Pyrroline-based cyclic nitrones such as 5,5-dimethyl-pyrroline N-oxide and 5-diethoxyphosphoryl-5-methyl pyrroline N-oxide are the most frequently utilized spin traps [281]. Both types of spin traps react with the ^•^OH and O_2_^•−^ radicals and form –OH and –OOH adducts, respectively. In comparison to other spectrophotometric techniques, EPR facilitates the interpretation of complex heterogeneous solutions [282]. However, the short lifetime of free radicals and the low-rate extent of radical generation in living tissues and organs make radical detection by EPR challenging. As a result, rapid-scan EPR has emerged as a powerful technique for sensitive detection of ROS with excessively short lifetimes (e.g., ^•^OH and O_2_^•−^) at ambient temperatures, which were difficult to detect using EPR [283].

It is a contentious issue to improve existing protocols and assays for assessing the antioxidant potency of nanoantioxidants. Assays based on the spectrophotometric or spectrofluorimetric basis are subjected to light scattering exhibited by a variety of nanomaterials. In this context, inhibited autoxidation methods, which rely on measuring the autoxidation rate of a reference substrate in the presence and absence of nanoantioxidants, are among those methods that cause less interference. Inhibited autoxidation methods are preferable protocols because they assess the antioxidant activity in close-to-real settings. Monitoring the absence of reactants, most commonly O_2_ or the generation of oxidation products (e.g., hydroperoxides or conjugated dienes) can be used to assess inhibited autoxidation kinetics [284]. Oximetry methods measure oxygen (O_2_) consumption and are carried out in closed systems and monitored by a fluorescence quenching probe [285], a polarographic probe [286], or a pressure transducer [287]. The trend of O_2_ uptake in oximetry methods typically follows a biphasic behavior, with an induction phase in which the antioxidant suppresses the autoxidation accompanied by a rapid rate of autoxidation. Oximetry methods can be implemented in homogeneous organic solutions, water-based solutions, and heterogeneous models such as liposomes or micelles. The potency of nanoantioxidants can be easily assessed using oximetry methods with a differential pressure transducer because this method is not affected either by the color of the colloidal solution or the incidence of the suspended NPs [288].

### 6.2. Other Limitations of Nanoantioxidant Applications

Nanomaterial agglomeration may also cause interference during antioxidant determination assays. The morphological structure, shape, surface charge, and type of nanomaterials affect the agglomeration tendency, which further affects the cellular uptake and ultimately affects the associated toxicity [289]. Some adjustments and strategies to avoid interference of conventional antioxidant assays and to provide more reliable measurements of the nanoantioxidant biological effects are described as follows [290]:

- Use of well-dispersed nanomaterials (not-agglomerated);
- Application of washing and separation steps (e.g., centrifugation, filtration, and precipitation);
- Use of appropriate conditions similar to those of biological systems;
- Application of concentrations below toxicity ranges.

Overall, a combination of more than one detection assay will provide a fully comprehensive and informative picture of the antioxidant capacity of nanomaterials.

Nanoantioxidant doses and their frequency vary greatly based on the levels of RONS in different inflammatory diseases and even within the several phases of the same inflammatory disease. Application of appropriate doses of nanoantioxidants for the control of intracellular RONS should be directed towards therapeutic applications without exacerbating any adverse clinical manifestations. Ideally, nanoantioxidants should significantly contribute to the well-being of patients and reduce or prevent treatment-induced health side-effects to overcome potential risks. Enhancing biocompatibility and feasibility of nanoantioxidants are also issues of concern that need to be addressed prior to commercialization of nanoantioxidants. Scientists have spent the previous decade assessing and monitoring the impact of nanomaterials on biological systems and it has been suggested that the cytotoxic effects of nanomaterials are variable and can be reduced [52].

From a synthesis point of view, optimizing the synthesis parameters would contribute to the development of high-quality nanoantioxidants and well-defined and reproducible nanostructures with better physicochemical characteristics. Cost-effective commercial production is also a crucial step that should be considered for the potential use of nanoantioxidants in biomedical practices. Long-term chronic cytotoxic effects need to be thoroughly investigated and assessed for safe in vivo applications. The precise identification of nature, physicochemical properties, and the mechanism of action of functionalized nanoantioxidants is a prerequisite for achieving the maximum biological benefits [54]. Additionally, a comprehensive assessment of the toxicity of non-biodegradable nanoantioxidants is a vital issue for any future biomedical application. Hence, applications of sophisticated safety measurements for precise analysis, assessment, and toxicity evaluation are the basic requirements. To address some of the toxicity issues, nanoantioxidants derived from various biological sources, such as bacteria, fungi, algae, plants, and lichens have emerged as significant alternative options for improved biocompatibility, durability, efficacy, and stability. Biological sources are efficient producers of a wide range of biomolecules and secondary metabolites [291]. The amalgamation of these biologically active compounds mediates the manufacture of biogenic-derived nanomaterials with antioxidant potency.

## 7. Future Insights of Nanoantioxidant Applications

Nanoantioxidants have exhibited high potency for oxidative stress depletion with high cellular antioxidant potential, specificity, and targeted delivery. However, to achieve significant clinical benefits, a thorough understanding of the detailed antioxidative mechanisms of nanoantioxidants necessitates extensive cross-collaboration of experts from various fields, including materials science, chemistry, as well as physical and biomedical fields. In-depth mechanistic studies of oxidative stress in vivo will be helpful to revolutionize the nanoantioxidant therapeutic field. Identification of the oxidative species that are primarily responsible for the onset and progression of oxidative stress-related diseases is required. Most notably, the use of nanoantioxidants will be significantly dependent on their rational design, characterization, as well as tools and methods that can provide a thorough and detailed understanding of their antioxidant activities. The tailored design of nanoantioxidants can result in significant antioxidative potential in vivo and, as a result, improve clinical effectiveness. It is necessary to precisely identify the nature, physicochemical properties, and mechanisms of action of nanoantioxidant composites in terms of their biological and catalytic activities. Searching for the best and the most cost-effective routes for the preparation of nanoantioxidants is necessary while minimizing their cytotoxic effects. The use of advanced models to assess their cytotoxic effects will aid in the robust evaluation of emerging antioxidative monotherapies. Prior to practical applications of nanoantioxidants, more research is required related to their in-situ delivery and on-time release at target sites. Evaluation of the benefits and side effects of using nanoantioxidants is necessary before they can be used safely in vivo, particularly for long-term treatments. Moreover, novel and efficient therapeutic nanoantioxidant delivery methods are required. Understanding the nature of new nanostructures with antioxidant activity will thus pave the way for nanoantioxidant-mediated treatments in the future.

To summarize, future studies shall be aimed at (i) analyzing the impact of prolonged exposure to minor concentrations of nanoantioxidants; (ii) assessing the potential risks of nanoantioxidants during fabrication, manipulation, and storage; (iii) simplifying the fabrication processes for the manufacture of highly potent nanoantioxidants; (iv) enhancing the reproducibility, biocompatibility, reliability, robustness, and stability of nanoantioxidants in biological microenvironments; and (v) designing microreactors that can effectively control the parameters of reaction synthesis.

## 8. Conclusions

Specific types of nanoantioxidants have emerged as pioneer types due to their antioxidant potency, demonstrating a superior potential to reduce oxidative stress with increased sensitivity, low cytotoxicity, and better-targeted delivery. Ceria-, silica-, PDA-, polysaccharide-, protein-, and nanocomposite-based nanoantioxidants are among the most interesting and investigated types of nanoantioxidants due to their unique physico-chemical properties, which can be tunned/adjusted to produce superior multifunctional nanoantioxidants. This can be accomplished by monitoring the preparation methods and optimizing the reaction parameters to obtain the desired types of nanoantioxidants with proper surface functionalization. Bioengineered nanomaterials have emerged as promising green nanoantioxidants and better alternatives due to their biocompatibility, biodegradability, low toxicity, and stability. Solid lipid NPs, nanostructured lipid carriers, and liposome NPs are efficient nanoencapsulation systems. The toxicity concerns of nanoantioxidant applications have been emphasized. The advantages, limitations, and future insights of nanoantioxidant applications have been proposed to achieve a win-win situation in the fight against oxidative stress-mediated disorders.

## Figures and Tables

**Table 1:** The most common methods applied for the preparation of nanoantioxidants.

Preparation Techniques	Suitable Materials	Advantages	References
Templating	Mesoporous materials	Easy operation, precise control over particle size, shape, and structure, less sensitive to operating conditions	[52]
Supercritical fluid	Temperature-sensitive materials	Mild operating conditions	[62]
Emulsion/solvent evaporation	Polymeric materials	Rapid, mild operating conditions, economical, no toxic solvents	[54]
Solvent displacement (Nanoprecipitation)	Lipophilic, polymeric, and bioactive materials	Facile, rapid, reproducible, formation of polymeric NPs, nanospheres, and nanocapsules	[63]

**Table 2:** List of biologically fabricated monometallic, bimetallic, and metal oxide NPs endowing antioxidant potential.

Type of NPs	Biological Extract	Size (nm), Shape	Characterization Techniques	Evaluation Assays	IC_50_ Value (µg/mL)	Antiradical/Antioxidant Activity (%)	References
Monometallic-Based Nanoantioxidants							
Ag	*Malus domestica*	100, spherical	UV/Vis spectroscopy, SEM, Zeta potential	DPPH	-	75.16	[173]
Ag	*Asphodelus aestivus*	50, spherical	UV/Vis spectroscopy, NTA, XRD, TEM, FTIR	DPPH, ABTS^•+^, H_2_O_2_	-	67.54, 79.94, 31.67	[174]
Ag	*Lippia* *nodiflora*	30–60, spherical	UV/Vis spectroscopy, FTIR, XRD, SE-EDX, TEM, Zeta potential	DPPH, O_2_^•−^, H_2_O_2_, ^•^OH	-	67, 70, 71.1, 69	[175]
Ag	*Memecylon umbellatum*	7–22, spherical	UV/Vis spectroscopy, FTIR, HRTEM, XRD, Zeta potential	DPPH	53.46	81.57	[176]
Ag	*Calophyllum tomentosum*	24, spherical	UV/Vis spectroscopy, FTIR, XRD, EDX, SEM	DPPH, H_2_O_2_, ^•^NO	-	90, 83.94, 78.46	[177]
Ag	*Morus alba*	12–39, spherical	UV/Vis spectroscopy, FTIR, HRTEM, XRD, FESEM, EDX	ABTS^•+^, DPPH, O_2_^•−^, ^•^NO	25.929, 97.273, 37.097, 70.992	95.08, 47.81, 81.92, 64.04	[178]
Ag	*Piper longum*	28.8, spherical	UV/Vis spectroscopy, FESEM, XRD, FTIR, HRTEM	DPPH	-	78.64	[179]
Ag	*Rosa canina*	19.75, spherical	UV/Vis spectroscopy, TEM, XPS, XRD	DPPH	-	86.4	[180]
Ag	*Clove bud*	17.94, spherical	UV/Vis spectroscopy, EDX, XRD, FTIR, Zeta potential, cyclic voltammetry	ABTS^•+^, ^•^OH	33.03, 47.37	-	[181]
Ag	*Cleome viscosa*	50, Rod/spherical/ triangular	UV/Vis spectroscopy, FTIR, XERD, SEM, TEM	DPPH, ABTS^•+^	20.32, 48.5	92.8 77.63	[182]
Ag	*Allium ampeloprasum*	35, spherical/quasi spherical/ ellipsoidal/ triangular/hexagonal	UV/Vis spectroscopy, FTIR, XRD, TEM	DPPH, ABTS^•+^, H_2_O_2_, ^•^NO, O_2_^•−^	8.93, 18.31, 11.25, 16.51, 23.22	-	[183]
Ag	*Aesculus* *hippocastanum*	50 ± 5, spherical	UV/Vis spectroscopy, FTIR, XRD, SEM, Zeta potential	DPPH, O_2_^•−^	-	93.48 62.9	[184]
Ag	*Lactobacillus brevis*	45, spherical	FTIR, XRD, SEM, TEM, elemental analyzer	DPPH, H_2_O_2_, ^•^NO	-	81.4, 70.1, 75.06	[185]
Ag	*Achillea millefolium*	20.77 (spherical), 18.53 (triangular), 14.27 (cubic)	UV/Vis spectroscopy, FTIR, SEM, XRD	DPPH	7.03	-	[186]
Ag	*Cannabis sativa*	11.5, spherical	UV/Vis spectroscopy, FTIR, FESEM, TEM	DPPH	218	-	[187]
Au	*Lotus leguminosae*	37, spherical	UV/Vis, IR, TEM, TGA	DPPH	30.54	-	[188]
Au	*Clove bud*	27.12, hexagonal/ polyhedral	UV/Vis spectroscopy, EDX, XRD, FTIR, Zeta potential, cyclic voltammetry	ABTS^•+^, ^•^OH	36.76, 50.32	-	[181]
Au	*Curcuma pseudomontana*	20, spherical	UV/Vis spectroscopy, SEM, HRTEM, FTIR	DPPH, H_2_O_2_, ^•^NO, reducing power, CUPRAC	-	85.2, 83.2, 84.5, 87.9, 85.6	[189]
Ag, Au	*Plumbago zeylanica*	28.47 (spherical), 16.89 (spherical)	UV/Vis spectroscopy, FTIR, TEM, XRD, EDX	DPPH	56.98 (Ag), 68.53 (Au)	78.17, 87.34	[190]
Pt	*Tragia* *involucrata*	10, spherical	UV/Vis spectroscopy, XRD, FTIR, FESEM, HRTEM	DPPH	-	64 ± 0.43	[191]
Cu	*Falcaria* *vulgaris*	20–25, spherical	UV/Vis spectroscopy, XRD, HRTEM, FESEM, FTIR	DPPH	190	-	[192]
Cu	*Borreria* *hispida*	121 ± 37, quasi spherical	UV/Vis spectroscopy, XRD, SEM, EDX, FTIR	DPPH	0.6	-	[193]
Bimetallic-Based Nanoantioxidants							
Ag/Cu	*Borassus* *flabellifer*	80, -	UV/Vis spectroscopy, FTIR, SEM	DPPH, ^•^OH, H_2_O_2_	-	58, 48, 42	[194]
Cu/Zn	*Borassus* *flabellifer*	100, -	UV/Vis spectroscopy, FTIR, SEM	DPPH, ^•^OH, H_2_O_2_	-	40, 38, 28	[194]
Ag/Pt	*Vernonia Mespilifolia*	35.5 ± 0.8, spherical	UV/Vis spectroscopy, TEM, EDX, FTIR	DPPH, ABTS^•+^, FRAP	19.5 21.6 44.1	-	[195]
Au/Ag	*Clove bud*	16.04, spherical	UV/Vis spectroscopy, EDX, XRD, FTIR, Zeta potential, cyclic voltammetry	^•^OH, ABTS^•+^	30.59 18.27	-	[181]
Metal Oxide-Based Nanoantioxidants							
CuO	*Cucurbita sp.*	45–65, rod/ rectangular/ hexagonal	UV/Vis spectroscopy, XRD, SEM, EDX, HRTEM	DPPH	40.81	91.37	[196]
MgO	*Pisonia alba*	<100, spherical	UV/Vis spectroscopy, TEM, EDX, XRD, FTIR	DPPH, FRAP	-	65, 69.3	[197]
ZnO	*Tecoma* *castanifolia*	70–75, spherical	UV/Vis spectroscopy, TEM, XRD, FTIR	DPPH	-	56.11	[198]
ZnO	*Knoxia* *sumatrensis*	50–80, rod	UV/Vis spectroscopy, XRD, FTIR, FESEM	DPPH, ABTS^•+^, H_2_O_2_	95.80, 92.29, 98.92	- 50.70 -	[199]
CuO	*Cissus* *vitiginea*	~32.3, spherical	XRD, EDS, TEM	DPPH	45.29	86.78	[200]
TiO_2_	*Cola nitida*	25–191, spherical	UV/Vis, FTIR, TEM, EDX, XRD, FESEM	DPPH, H_2_O_2_	-	62.06 99.23	[201]

**Table 3:** Composition and characteristic features of each liposomal new generation.

Type	Composition	Advantages	References
Transfersomes	Lipid chains and surfactants “edge activators”	High elasticity, flexibility, and penetration potential	[243,244]
Ethosomes	Phospholipids, water, and high ethanol concentrations (20–45%)	High elasticity, permeability, distribution, flexibility, steric stability, and low aggregation	[245]
Niosomes	Non-ionic surfactant	Ease of production, low production cost, high chemical stability and storage	[246]
Cubosomes	Lipid cubic phase and coated by apolymer-based outer coating	High surface area, water solubility, better stability, and encapsulation efficiency	[247]
Biosomes	Bile salts	Deep penetration, high stability, and encapsulation efficiency	[248]
Novasomes	Numerous amphiphiles such as fatty alcohols and acids with each other, or with phospholipids	Precise targeting and efficient delivery	[249]
Vesosomes	Liposomal vesicles encapsulating smaller liposomes of different sizes	High protection and encapsulation efficiency	[250]
